# Cryo-EM advances in RNA structure determination

**DOI:** 10.1038/s41392-022-00916-0

**Published:** 2022-02-23

**Authors:** Haiyun Ma, Xinyu Jia, Kaiming Zhang, Zhaoming Su

**Affiliations:** 1 The State Key Laboratory of Biotherapy, Department of Geriatrics and National Clinical Research Center for Geriatrics, West China Hospital, Sichuan University, Chengdu, Sichuan 610044 China; 2 MOE Key Laboratory for Cellular Dynamics and Division of Life Sciences and Medicine, University of Science and Technology of China, Hefei, 230027 China

**Keywords:** Structural biology, Non-coding RNAs, Riboswitches, Structural biology

## Abstract

Cryo-electron microscopy (cryo-EM) has emerged as an unprecedented tool to resolve protein structures at atomic resolution. Structural insights of biological samples not accessible by conventional X-ray crystallography and NMR can be explored with cryo-EM because measurements are carried out under near-native crystal-free conditions, and large protein complexes with conformational and compositional heterogeneity are readily resolved. RNA has remained underexplored in cryo-EM, despite its essential role in various biological processes. This review highlights current challenges and recent progress in using cryo-EM single-particle analysis to determine protein-free RNA structures, enabled by improvement in sample preparation and integration of multiple structural and biochemical methods.

The recent resolution revolution triggered by the development of direct electron detectors and other technical advances has allowed cryo-EM to break the previous resolution barrier(s) and led to exponential growth of near-atomic cryo-EM structures.^1,2^ More recently, several studies have achieved atomic-resolution cryo-EM reconstruction using either the next-generation hardware or the most up-to-date commercially available setup.^3–5^ Aside from single-particle analysis (SPA) that has gained tremendous popularity, other cryo-EM approaches have also benefited from the revolution, such as cryo-electron tomography (cryo-ET),^6,7^ micro-electron diffraction (MicroED)^8^, and cryo-scanning transmission electron microscopy (cryo-STEM).^9^ Because cryo-EM SPA is featured by minimal amount of specimen under near-native crystal-free condition, automated data collection with continually increasing throughput,^10^ and comprehensive data processing pipelines capable of resolving structural heterogeneity,^11^ it has become a widely adopted structural biology technique for structural biologists whose portfolio used to have only X-ray crystallography and NMR. Whereas the above features make SPA particularly useful to determine structures of larger proteins and protein complexes (>200 kDa), additional technical advances, especially the development of Volta phase plate (VPP),^12^ have pushed the limit of protein molecular weight to as low as 52 kDa for structure determination by SPA.^13^ Readers interested in technical developments and current challenges in SPA are directed to a number of comprehensive reviews for further details.^11,14–16^

RNA plays an essential role in various important biological processes by folding and sustaining a three-dimensional (3D) structure in order to perform functions such as catalysis and gene regulation.^17–19^ It is estimated that about 85% of the human genome is transcribed into RNA,^20^ with more than 80% of the genome estimated to be biologically and functionally relevant,^21^ however, only 1.5 percent of the genome encodes proteins.^21,22^ In the Protein Data Bank (wwPDB), there are currently 183980 depositions of protein complexes, which has facilitated the development of accurate protein structure prediction algorithms.^23^ In contrast to our apparently better understanding of protein structures and functions, our knowledge of RNA structures remains scarce. There are currently 1569 protein-free RNA and 9790 protein-nucleic acid complex structures deposited in the PDB that accounts for only about 6% of the entire PDB deposition (Fig. 1a). The lack of RNA structures is most likely due to the intrinsic heterogeneity of RNAs caused by flexible ribose and phosphate backbone, weak long-range tertiary interactions, alternative conformations, and dynamics among multiple functional states, which pose great challenges to X-ray crystallography to obtain RNA crystals with high-resolution diffraction information, and to NMR to solve structures of RNAs larger than 100 nucleotides (100 nt).^24,25^ Although cryo-EM SPA has extended our accessibility to more challenging biological structures and systems, protein-free RNA structures accrue at a much slower rate compared to proteins and protein-nucleic acid complexes in the Electron Microscopy Data Bank (EMDB). To date, there are only three protein-free RNA cryo-EM structures determined at 4 Å or better resolution (Table 1), which accounts for 0.02% of the entire 17349 depositions in EMDB (Fig. 1b).

**Fig. 1:**
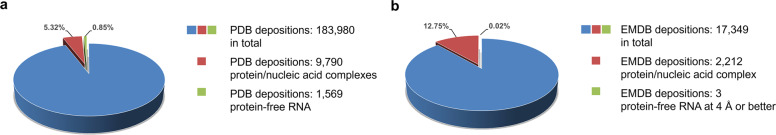
Statistics of protein-free RNA and protein-nucleic acid complex structures in PDB (**a**) and EMDB (**b**)

**Table 1:** Summary of RNA cryo-EM structures better than 10 Å resolution

RNA specimen (reference)	*M*_w_ (nt/kDa)	#particles in 3D reconstruction	Resolution (Å)	VPP	Cryo-EM *B*-factor (Å^2^)
ATP-TTR-3 (with AMP)^37^	130/42	39,136	10	Yes	1398
ATP-TTR-3 (apo)^37^	130/42	71,045	10	Yes	1501
*F. nucleatum* glycine riboswitch (with glycine)^37^	171/55	35,578	7.4	Yes	415
*F. nucleatum* glycine riboswitch (apo)^37^	171/55	20,269	10	Yes	840
hc16 ligase^37^	338/108	21,236	10	Yes	1002
hc16 ligation product^37^	349/112	29,191	10	Yes	1055
*Tetrahymen*a L-21 ScaI ribozyme (apo)^37^	388/125	74,621	6.8	Yes	553
HIV-1 DIS^45^	94/30	24,934	9	No	N/A
*Mycobacterium* SAM-IV riboswitch (with SAM)^37^	119/40	225,303	4.8	No	303
*Mycobacterium* SAM-IV riboswitch (apo)^37^	119/40	260,244	4.7	No	238
*Mycobacterium* SAM-IV riboswitch (with SAM)^41^	119/40	588,580	4.1	No	314
*Mycobacterium* SAM-IV riboswitch (apo)^41^	119/40	796,923	3.7	No	219
*V. cholerae* glycine riboswitch (with glycine)^37^	171/55	193,317	5.7	No	393
*V. cholerae* glycine riboswitch (apo)^37^	171/55	230,891	4.8	No	317
*B. subtilis* glyQS T-box (with tRNA^Gly^)^46^	244/74	189,361	4.9	No	232
*L. lactis* L1 LtrA-depleted intron RNA^33^	702/216	102,522	4.5	No	N/A
dENE^49^	76/23	69,623	8.7	No	N/A
dENE-poly(A_28_)^49^	104/32	283,486	5.6	No	N/A
*Tetrahymena* L-21 ScaI ribozyme (apo)^42^	388/118	415,918	3.1	No	126
*Tetrahymena* L-16 ScaI ribozyme with two RNA oligonucleotide substrates^42^	407/125	230,386	3.1	No	90
SARS-CoV-2 FSE^44^	88/28	109,137	5.9	No	726
BMV TLS^35^	169/55	128,266	4.3	No	N/A
*Tetrahymena* group I intron^36^	388/118	82,575	3.0	No	N/A
*Azoarcus* group I intron^36^	197/60	486,860	4.9	No	N/A
*F. nucleatum* FMN riboswitch^36^	112/34	266,623	5.9	No	N/A

The scarcity of protein-free RNA cryo-EM structures may be attributed to the following reasons: (1) Effective approaches and/or pipelines are missing for obtaining properly folded RNAs with stable tertiary structures; (2) the majority of isolated functional RNAs are simply too small for facile visualization and reconstruction in standard SPA pipelines; (3) intrinsic heterogeneity in most RNA molecules greatly limits the attainable resolution by SPA. This review summarizes recent efforts that overcome these challenges and enrich the protein-free RNA cryo-EM structure repertoire, including new approaches to obtain stable RNA structures for cryo-EM SPA and integration of multiple techniques to facilitate structural analysis when resolution is moderate. Cryo-EM structures of RNAs in RNPs such as ribosome, spliceosome, telomerase, and CRISPR complexes will not be discussed, as they are reviewed elsewhere.^26–29^ All RNA structures reviewed herein are cryo-EM structures of protein-free RNAs.

## New approaches to obtain stable RNA structures for SPA

**Fig. 2:**
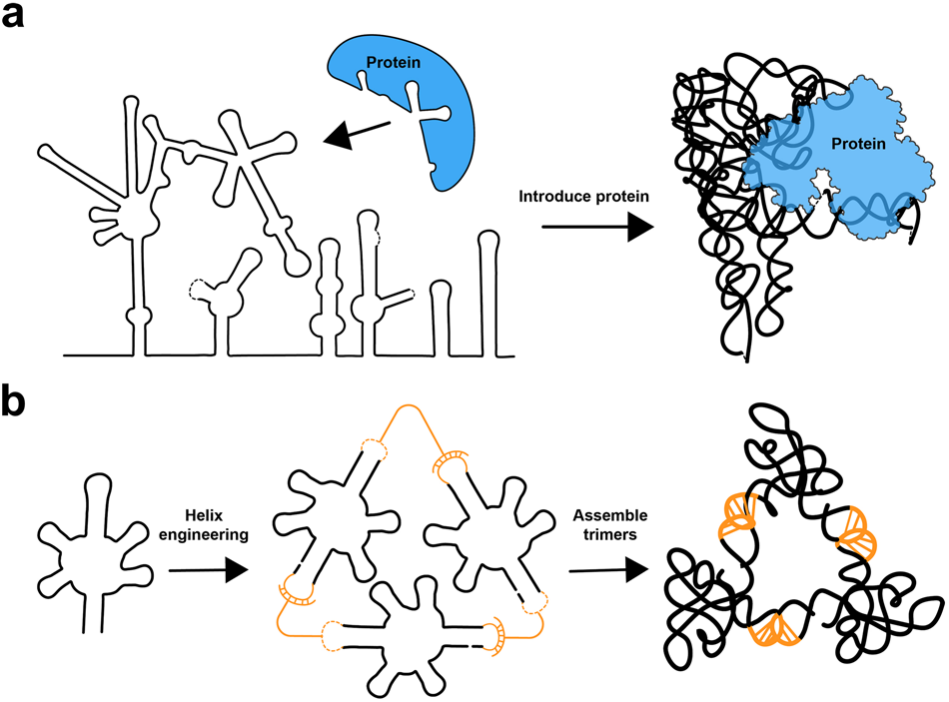
New approaches to obtain stable RNA structures for cryo-EM SPA. (**a**) Protein binding partners can be introduced to stabilize RNA structures. (**b**) Self-assembled homomeric RNA nanostructures potentially allow cryo-EM SPA studies of relative small RNAs

RNA X-ray crystallography utilizes strategies that introduce proteins such as U1A and L7Ae to bind to specific RNA motifs to stabilize RNA crystal lattices.^30,31^ Group II introns are ribozymes with endogenous protein partners that stabilize the dynamic RNA structures and facilitate RNA catalysis.^32,33^ The analogous approach has been reported by Qu et al. in the cryo-EM structure of the Lactococcus lactis LtrB group II intron in complex with its intron-encoded protein LtrA at 3.8 Å (Fig. 2a), in which they utilize a 60 kDa RNA binding protein LtrA to stabilize the RNA structure and resolve 704 nt (216 kDa) of the 902 nt LtrB using cryo-EM SPA.^34^ Intriguingly, 3D classification yielded one minor class that resembled the LtrB intron alone with four times fewer particles than the LtrB-LtrA RNP, which generated a protein-free RNA structure at 4.5 Å. The disordered LtrA binding site in LtrA-depleted RNA structure validates the role of LtrA to assist folding and to enhance the stability of LtrB intron structure as previously described.^34,35^

Bonilla and colleagues used cryo-EM SPA to solve the viral tRNA-like structure (TLS) of bromo mosaic virus (BMV) at 4.3 Å resolution.^36^ Unexpectedly, the “tRNA-like” L-shape was not readily observed in protein-free BMV TLS. Comparison of the TLS alone with the complex structure of TLS and tyrosyl-tRNA synthetase (TyrRS) indicates that TLS undergoes drastic conformational changes to resemble “tRNA-like” shape in order to bind to TyrRS. This is a great example illustrating the importance of studying protein-free RNAs to provide insights of required conformational rearrangement upon protein binding.

Liu and coworkers recently reported a nanostructure assembly strategy called “RNA oligomerization-enabled cryo-EM via installing kissing-loops (ROCK)”, to obtain homomeric self-assembled dimers and trimers of protein-free RNAs for SPA at near-atomic to subnanometer resolutions.^37^ Self-assembly is achieved by insertions of kissing-loop sequence in the functionally non-essential peripheral stem loops, which makes the assembled RNA two- to three-times larger than the monomeric RNA (Fig. 2b). While this strategy could be potentially useful for RNAs too small for particle picking and orientation alignment in cryo-EM SPA, it may also have an artificial impact on RNA dynamics after oligomerization. Prior knowledge of RNA structure-function relationship is also required to guide insertions of kissing-loop sequences.

## New workflow that accelerates the determination of RNA cryo-EM structures

**Fig. 3:**
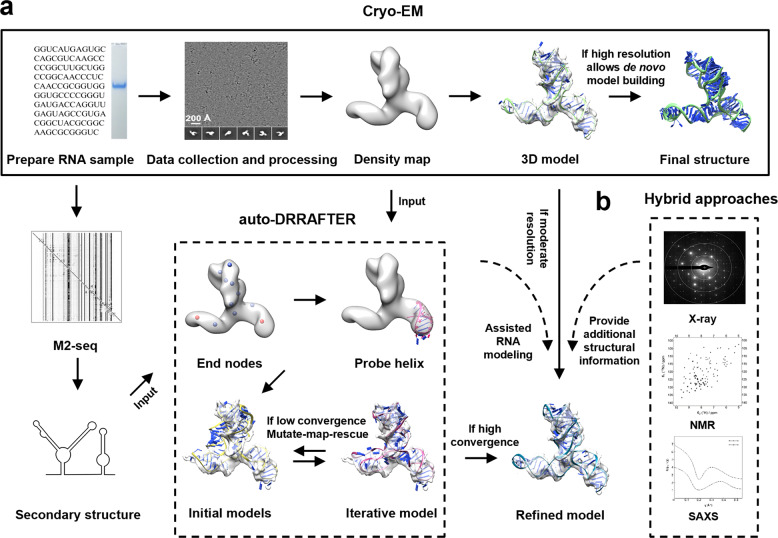
Cryo-EM-guided RNA structure determination. **a** “Ribosolve” utilizes native gel analysis, secondary structure information, cryo-EM maps at moderate resolution, and RNA modeling algorithm to generate RNA models. **b** Cryo-EM maps at moderate resolution that do not allow de novo modeling can be combined with structural information determined by other techniques like X-ray crystallography, NMR and SAXS

Kappel et al. recently developed an accelerated RNA structure determination workflow, named “Ribosolve” (Fig. 3a), that integrates native gel analysis, mutate-and-map by next generation sequencing (M2-seq), cryo-EM SPA, and auto-DRRAFTER RNA modeling.^38^ In this workflow, native gel analysis enables quick identification of optimal RNA refolding conditions. RNAs that form sharp bands in native gels are subjected to M2-seq experiments,^39^ in which secondary structures are determined to both assess the homogeneity of folded RNA structures and to assist RNA modeling. Cryo-EM SPA allowed the rapid determination of 11 previously unknown RNA structures at resolution ranging from 4.7 to 10 Å, which revealed explicit RNA features of major and minor grooves. Seven structures were determined with VPP for contrast enhancement. Finally, auto-DRRAFTER was developed based on the previous DRRAFTER^40^ to utilize secondary structures determined by M2-seq to build 3D RNA models guided by cryo-EM density, because *de novo* RNA modeling can be very challenging under such resolution. The estimated coordinate root mean square deviation (RMSD) accuracy was between 3.3 and 6.3 Å as predicted by modeling convergence, which is sufficient for revealing RNA overall fold. This workflow enables accelerated RNA cryo-EM structure determinations by introducing methods to examine RNA folding and structural homogeneity, which can be used both to assist RNA modeling and to guide additional cryo-EM data collection for improved resolution.

A few applications of the “Ribosolve” workflow have been reported. SAM-IV riboswitch, a 40 kDa RNA that recognizes S-adenosylmethionine (SAM),^41^ was initially resolved to 7 Å resolution using cryo-EM SPA with VPP. Zhang et al. continued to collect additional cryo-EM data and achieved 3.7 Å resolution for the apo state and 4.1 Å for the SAM-bound state allowing unambiguous ligand recognition.^42^ The final data sets that generated cryo-EM structures of both states at near-atomic resolution were collected without VPP. This is likely because the stronger electron scattering of phosphate backbones has provided sufficient contrast for visualization of this small RNA. At 3.7 Å resolution, only a portion of the base pairs is resolved, indicating that higher resolution is required to allow *de novo* modeling. Nonetheless, this study has demonstrated the capability of cryo-EM SPA to facilitate structure determination at near-atomic resolution for RNA that is beyond the current molecular weight limit of 52 kDa for protein.^13^

Recently, Su and colleagues solved the cryo-EM structures of full-length *Tetrahymena* ribozyme in both apo and substrate-bound states at 3.1 Å resolution.^43^ Group I self-splicing intron of *Tetrahymena thermophila* was discovered as the first ribozyme and has since become an unprecedented model system to study RNA catalysis and structure-function relationship. However, the full-length *Tetrahymena* ribozyme structure remains unknown for 40 years. After a 6.8 Å cryo-EM map of the apo L-21 *Tetrahymena* ribozyme map was obtained by “Ribosolve”, more data was collected to achieve a 3.1 Å cryo-EM structure, in which the complete peripheral region was explicitly resolved and two previously unforeseen tertiary interactions that allosterically regulate catalysis were identified. The substrate-bound state revealed catalytic mechanism, conformational changes of the internal guide sequence, and local shifts of phosphate, nucleobase, and metal ions upon substrate binding. This study provided a complete structural view of the *Tetrahymena* ribozyme and a great example of dissecting the RNA structure-function relationship by cryo-EM.

During the SARS-CoV-2 pandemic, cryo-EM SPA has played essential roles in unveiling the structural basis of viral infection, replication, and other stages in the virus life cycle.^44^ Using “Ribosolve”, Zhang et al. resolved the 6.9 Å cryo-EM structure of the frameshifting stimulus elements (FSE), a highly conserved 88 nt RNA that is essential for the balanced expression of important viral proteins in the SARS-CoV-2 genome.^45^ It is also a potential candidate target for antiviral drugs such as small molecules and antisense oligonucleotides (ASO). Under the guidance of cryo-EM map, the RNA model was built with an estimated RMSD accuracy of 5.9 Å predicted by RNA modeling convergence following the “Ribosolve” pipeline.^38^ Subsequently, ASOs targeting FSE were designed and their antiviral activities were verified in vitro and at cellular level. This study features the smallest 28 kDa protein-free RNA studied by cryo-EM and resolving the tertiary structure of this key RNA element of SARS-CoV-2 potentially speeds up the development of new therapies for COVID-19.

## Hybrid approaches to facilitate RNA structural analysis

Zhang et al. used cryo-EM SPA to resolve a 30 kDa HIV-1 dimer initiation site RNA (DIS) at 9 Å resolution. The cryo-EM map helps restraining NMR model refinement to derive atomic ensembles of this RNA duplex to identify a flipped-out nucleotide at both ends of the duplex.^46^ Molecular dynamics (MD) simulations of multiple time points on simulated cryo-EM data of DIS suggest that the intrinsic RNA structural heterogeneity limits cryo-EM SPA from achieving higher resolution for the DIS RNA.

Li et al. resolved a 4.9 Å cryo-EM structure of the full-length T-box-tRNA^Gly^ complex.^47^ Although the central tRNA^Gly^ is better resolved to 4.1 Å, featured by separated density of some phosphate and ribose groups, the outer T-box is only resolved to 6 Å. This moderate resolution poses great challenge for RNA modeling, especially in the newly identified 66 nt 3′-discriminator region with previously unknown structural features. They tackled this problem by determining a co-crystal structure of tRNA^Gly^ in complex with the 3′-discriminator from another bacterial species at 2.7 Å resolution. The final model of the full length T-box-tRNA^Gly^ complex was generated by combining the 3′-discriminator RNA model with the previously known crystal structure of tRNA^Gly^ and 5′-Stem I complex and refined into the cryo-EM density.^48,49^ Cryo-EM 3D classification indicated that structural heterogeneity of the T-box 5′-Stem I and 3′-discriminator regions may have limited the overall resolution.

In another study, Torabi et al. resolved cryo-EM maps of a double element for nuclear expression (dENE) before and after poly(A) binding at subnanometer resolution.^50^ The cryo-EM structures show an overall similar architecture compared to a previous high-resolution crystal structure except for an ~23° curvature at the end of one ENE, indicating that crystal packing may not reveal minor structural dynamics in solution. Small angle X-ray scattering (SAXS) reveals a global shape that is consistent with both cryo-EM and crystal structures.

In conclusion, these examples illustrate the combinations of different high-resolution structural biology techniques, such as X-ray crystallography and NMR, to facilitate the analysis of cryo-EM RNA structures at moderate resolution (Fig. 3b). According to MD simulations and SPA 3D classification, the intrinsic structural heterogeneity is the major factor that limits resolution in cryo-EM reconstructions of RNA molecules.

## Perspective

Cryo-EM SPA can readily attain near-atomic resolution (between 2 and 4 Å) for proteins and protein complexes on a regular basis, which is sufficient to enable *de novo* model building. In contrast, RNA cryo-EM structures at near-atomic resolution are rare. The recent progress in RNA cryo-EM SPA has generated most RNA structures at a moderate resolution ranging from 4 to 10 Å, which are challenging for *de novo* modeling. Multiple strategies have been used synergistically to assist structural analysis of these RNA cryo-EM structures, including hybrid approaches combining RNA modeling, NMR, and X-ray crystallography. In order to facilitate structural analysis based on cryo-EM structures, it is critical to identify and overcome the major factors that limit cryo-EM SPA to attain RNA structures at near-atomic resolution.

In cryo-EM SPA, an overall temperature factor, or *B*-factor, derived from the correlation between the number of particles used for reconstruction and the achieved resolution, is used to evaluate the quality of the cryo-EM data (i.e. low *B*-factor indicates high data quality because fewer particles are required to achieve certain resolution).^51^ The *B*-factor values calculated for RNAs between 40 and 125 kDa are all greater than 200 Å^2^ (Table 1), whereas sub-2 Å cryo-EM structures of proteins normally have a *B*-factor around 60 Å^2,^^52^. Interestingly, *B*-factor values seem much larger for RNA structures reconstructed from cryo-EM data with VPP than those without VPP, indicating that VPP does not seem to help reducing the *B*-factor for RNA cryo-EM structures, albeit it significantly improves image contrast.

Biological samples are susceptible to radiation damage and limited electron exposure is used in Cryo-EM that leads to low contrast and signal-to-noise ratio (SNR), which will deteriorate the accuracy of particle alignment in SPA.^53,54^ The optimal exposure of cryo-EM SPA for protein has been determined to be ~20 e^−^/Å^2^, although higher dose could be used when exposure filtering is applied to enhance SNR at lower spatial frequencies in order to minimize alignment errors of particle orientations.^55^ Fujiyoshi et al. have previously found that tRNA crystals embedded in glycerol are at least 4 times less susceptible to radiation damage compared to protein crystals in glycerol based on the critical dose curve when exposed to electrons at room temperature.^56^ The radiation damage effect on RNAs embedded in vitrified ice under cryogenic temperature has not been characterized in detail. As more RNA cryo-EM structures are determined with continuously improving resolution, radiation damage and optimal exposure on RNA cryo-EM structures need to be assessed in order to guide optimal electron dose in cryo-EM data collection. For example, if RNA is less susceptible to radiation damage, higher total electron doses could be used in RNA cryo-EM SPA to enhance image contrast and SNR which will lead to attenuation of alignment errors. This may reduce the *B*-factor since small particles like RNAs are particularly sensitive to alignment errors.^57^

A number of other factors may have an impact on the *B*-factor, such as ice thickness, beam-induced motion (BIM), and intrinsic heterogeneity of the RNA molecules. To eliminate ice thickness variations, in which thicker ice will lead to decreased image contrast and SNR, novel sample preparation instruments have been developed and are currently optimized to reproducibly generate thin ice. For example, “Spotiton” can deliver as little as a few nanoliters of samples onto the grid while it plunges.^58^ The reduced time window between spotting and plunging also attenuates the air-water interface problem,^59^ which causes other issues in SPA such as preferred orientation and sample denaturation (reviewed elsewhere^60^).

BIM has been previously shown to be drastically reduced by the implementation of various supporting films and foils.^61^ In particular, the amorphous nickel-titanium alloy (ANTA) film has been developed to reduce BIM.^62^ More recently, BIM has been minimized utilizing ultrastable gold foils on gold grids with specific hole diameter that ameliorate stress in the vitrified ice, named HexAuFoil.^63,64^ The drastic translational and rotational motions discovered in the first few frames that carry the highest resolution information can also be ameliorated by finer intervals (i.e. more frames) to improve motion correction accuracy. The resulting lower contrast and SNR of each frame can be potentially compensated by VPP or weighted denoising algorithms that should theoretically enhance SNR at low spatial frequencies.^65,66^

RNA may naturally adopt higher *B*-factor than protein due to its intrinsic heterogeneity and dynamics. Structural study is primarily facilitated by the identification of RNAs with conformational homogeneity and stable 3D structures, as demonstrated by native gel analysis and M2-seq in the “Ribosolve” workflow.^37^ Additional information from computational analysis, such as phylogenetic analysis and predictions of long-range tertiary interactions (e.g. pseudoknot formations, minor-groove interactions, etc.) will further enable the identification of conformational homogeneous RNAs with stable 3D structures.^17,67^ It is noteworthy that cryo-EM provides the unique opportunity of resolving conformational heterogeneity. Recent advance in automated data collection strategies that uses beam-shift instead of mechanical stage movement for each exposure,^68^ combined with image processing algorithm that compensates the aberrations caused by beam-shift,^69^ enable significantly increased throughput of data collection. As more particles are collected within the same time frame, classification of multiple conformations become realistic using existing algorithms such as Relion multibody refinement,^70^ cryoSPARC 3D variability analysis,^71^ and the emerging neural network-based 3D heterogeneity analysis,^72^ to eventually resolve RNA structures and their intrinsic heterogeneity and dynamics.

We are in an exciting era for structural biology, in which the emerging deep learning structure prediction algorithms have enabled reliable structure predictions of proteins and protein complexes in eukaryotes including human.^23,73–76^ However, these algorithms rely heavily on the plentifulness of experimental data. Although a recent study has reported a scoring system trained by very few existing RNA structures using machine learning algorithm to improve RNA structure prediction accuracy,^77^ the lack of experimental RNA structures remains to be the major barrier for precise RNA structure prediction. Recent progress in the field of RNA cryo-EM demonstrates how cryo-EM SPA complements X-ray crystallography and NMR to provide insightful structural information. Continued advances in technical and experimental developments of cryo-EM will allow accelerated structural findings previously not accessible by X-ray crystallography and NMR, and generate RNA structures at near-atomic resolution on a more regular basis. This will provide structural insights to deepen our understanding of RNA structure-function relationships as well as facilitating precise RNA structure predictions in the near future.
